# Supplementary material for: Ni-Catalyzed Enantioselective Intramolecular Mizoroki–Heck Reaction for the Synthesis of Phenanthridinone Derivatives
Source: J Org Chem. 2023 Jun 15;88(13):8203–26. doi: 10.1021/acs.joc.3c00202 (PMC10337041; doi:10.1021/acs.joc.3c00202)
Supplement: Supplementary file 2 — jo3c00202_si_002.zip [file jo3c00202_si_002.zip › Heck products/2o Et-NMOM-pyridine Heck C4_N/13C Et-NMOM-pyridine Heck C4_N/3/pdata/1/email_DR-117-052 pure C13_3_1.pdf]

DR-117-052 pure C13
C13APT CDCl3 {D:\nmrusers\malachowski} BM 20

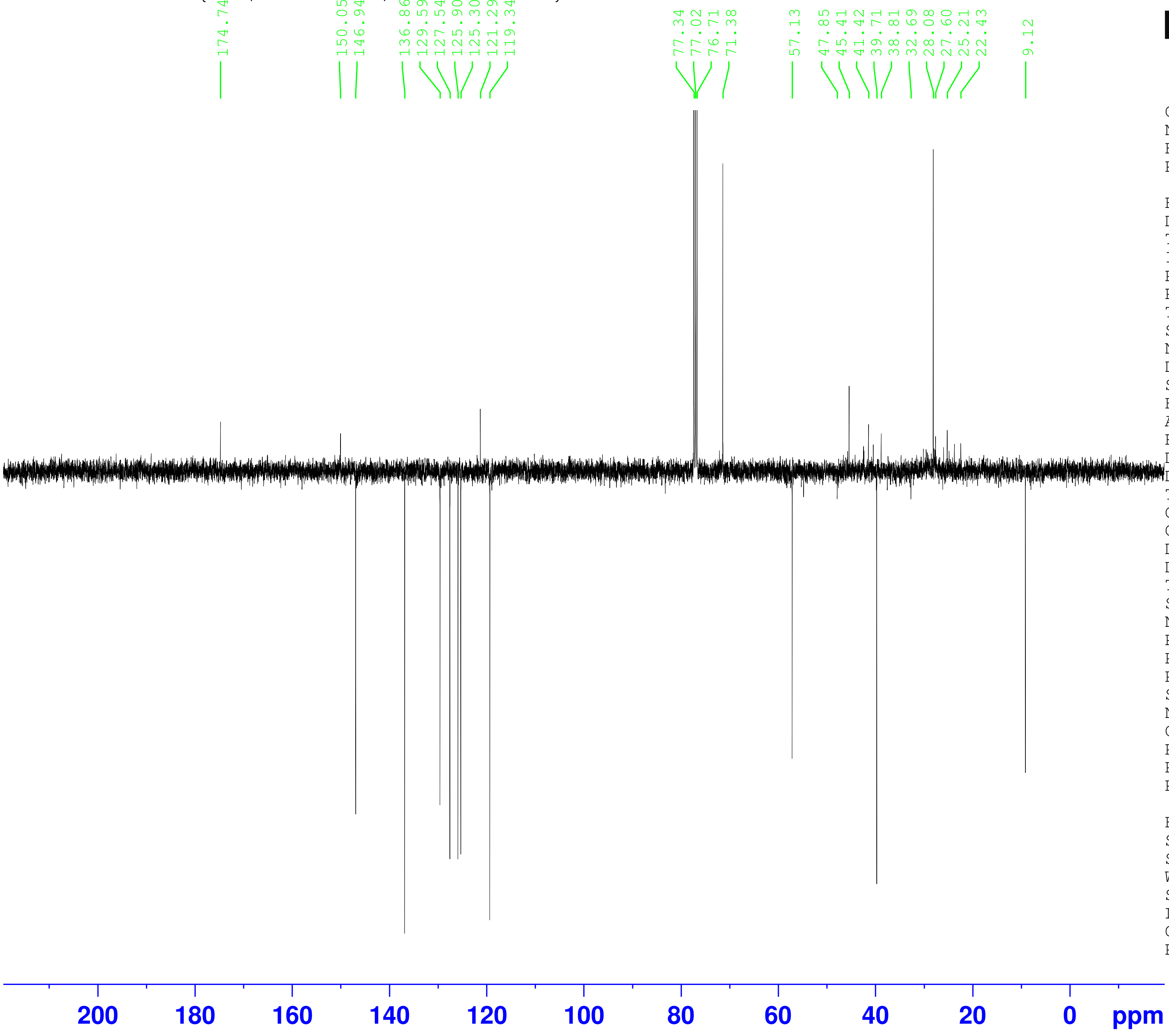


Current Data Parameters

| | |
|---|---|
| NAME | DR-117-052 pure C13 |
| EXPNO | 3 |
| PROCNO | 1 |

F2 - Acquisition Parameters

| | |
|---|---|
| Date_ | 20220707 |
| Time | 21.55 h |
| INSTRUM | spect |
| PROBHD | Z104450_0352 ( |
| PULPROG | jmod |
| TD | 65536 |
| SOLVENT | CDCl3 |
| NS | 4000 |
| DS | 4 |
| SWH | 24038.461 Hz |
| FIDRES | 0.733596 Hz |
| AQ | 1.3631488 sec |
| RG | 203 |
| DW | 20.800 usec |
| DE | 6.50 usec |
| TE | 298.0 K |
| CNST2 | 145.0000000 |
| CNST11 | 1.0000000 |
| D1 | 2.00000000 sec |
| D20 | 0.00689655 sec |
| TD0 | 1 |
| SFO1 | 100.6278593 MHz |
| NUC1 | 13C |
| P1 | 8.00 usec |
| P2 | 16.00 usec |
| PLW1 | 56.13299942 W |
| SFO2 | 400.1516006 MHz |
| NUC2 | 1H |
| CPDPRG[2 | waltz65 |
| PCPD2 | 90.00 usec |
| PLW2 | 12.14200020 W |
| PLW12 | 0.24368000 W |

F2 - Processing parameters

| | |
|---|---|
| SI | 32768 |
| SF | 100.6177975 MHz |
| WDW | EM |
| SSB | 0 |
| LB | 1.00 Hz |
| GB | 0 |
| PC | 1.40 |
